# Nonnutritive, Low Caloric Substitutes for Food Sugars: Clinical Implications for Addressing the Incidence of Dental Caries and Overweight/Obesity

**DOI:** 10.1155/2012/625701

**Published:** 2012-02-22

**Authors:** Michael W. Roberts, J. Timothy Wright

**Affiliations:** Department of Pediatric Dentistry, School of Dentistry, University of North Carolina, 228 Brauer Hall, CB No. 7450, Chapel Hill, NC 27599-7450, USA

## Abstract

Caries and obesity are two common conditions affecting children in the United States and other developed countries. Caries in the teeth of susceptible children have often been associated with frequent ingestion of fermentable sugars such as sucrose, fructose, glucose, and maltose. Increased calorie intake associated with sugars and carbohydrates, especially when associated with physical inactivity, has been implicated in childhood obesity. Fortunately, nonnutritive artificial alternatives and non-/low-caloric natural sugars have been developed as alternatives to fermentable sugars and have shown promise in partially addressing these health issues. Diet counseling is an important adjunct to oral health instruction. Although there are only five artificial sweeteners that have been approved as food additives by the Food and Drug Administration (FDA), there are additional five non-/low caloric sweeteners that have FDA GRAS (Generally Recognized as Safe) designation. Given the health impact of sugars and other carbohydrates, dental professionals should be aware of the nonnutritive non-/low caloric sweeteners available on the market and both their benefits and potential risks. Dental health professionals should also be proactive in helping identify patients at risk for obesity and provide counseling and referral when appropriate.

## 1. Introduction

It is estimated that 16–42% of Americans have untreated dental caries [1]. Recently, the prevalence of overweight children and adults has also increased, and diet choices affect both development of caries and contribute to weight gain. Recent prevalence data estimate that overweight among children has more than tripled since 1970 and affects 32% of all children and adolescents [2–7]. Excess body fat is the product of ingesting too many calories and reduced physical activity [8]. Obesity is a complex issue and no one cause has been identified. But, the consumption of high-sugar, low-nutrient foods is of particular concern [9–11]. It is generally accepted that sugars and prepared starches in the diet are significant contributors to these two health issues [12–18]. Frequency of exposure to the teeth and food retention are important considerations when evaluating the caries potential of food products [14]. Sucrose, fructose, and maltose are sugars commonly used in beverages and food products and add about 4 calories per gram. Consumption in developed countries is reported to be 40–60 Kg/person/year [19]. 

 Dentists and other oral health professionals have been active in counseling their patients regarding general health issues, such as monitoring blood pressure, smoking and alcohol cessation, and detection of child abuse/neglect. In addition, dentists have collaborated with medical colleagues in drafting guidelines for referring children with early childhood caries for definitive care, application of fluoride varnish and sedation. It has now been suggested that the oral health provider become an active participant in screening their patients, especially children, for signs of overweight/obesity and offer appropriate counseling/referral [12]. Other measurement tools, such as waist circumference, may be more accurate indicators of obesity, but the body mass index (BMI) is the most convenient screening means [20, 21]. This clinical tool measures body weight adjusted for height. Standardized BMI charts to determine BMI percentiles are available [22]. A “healthy weight” is described being below the 85th percentile.

 Industry and scientists have long searched for alternative sweeteners. The ideal product would have few or no calories, be noncarcinogenic or mutagenic, be economical to produce, and would not be heat degradable but would provide sweetness and have no unpleasant aftertaste. Obtaining these properties in a single product has been challenging. Numerous nonnutritive sweetening agents have been developed, but none have possessed all of the preferred properties.

 Patients and parents/care givers of children often ask oral health professionals about common dietary sugars and alternative sweeteners. The purpose of this paper is to provide the dental professional information that will be helpful in counseling these individuals on diet, caries prevention, and weight control [23].

## 2. FDA Approved Nonnutritive Sweeteners as Additives

**Figure 1:**
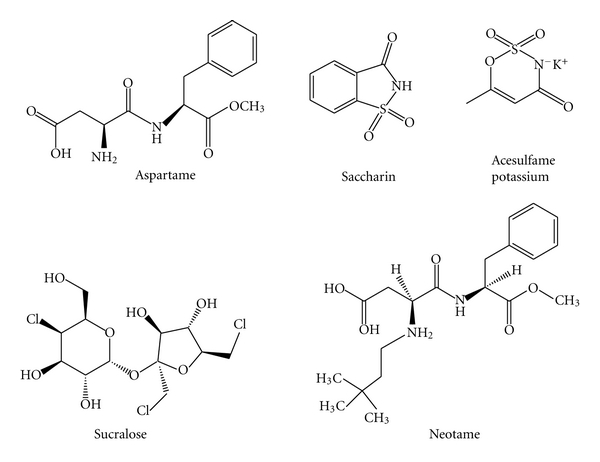
Chemical structure of FDA approved non-/low caloric sweeteners as food additives.

 Currently, the Food and Drug Administration (FDA) of the United States has evaluated the data and other information and approved under the conditions of its use only aspartame, acesulfame potassium, saccharin, sucralose, and neotame as noncaloric sweeteners as “food additives,” Figure 1. However, the FDA can also approve an agent under *GRAS *(Generally Recognized as Safe). For approval under *GRAS*, supporting data must have been provided and evaluated by qualified experts, and there is consensus that the substance is safe under the conditions of its intended use.

### 2.1. Saccharin

 Saccharin, first developed in 1878, is the oldest approved artificial sweetener. Initially granted *GRAS* status, saccharin is now approved as an additive to food and beverages. It is 300 times as sweet as sucrose by weight, non-cariogenic and noncaloric but can have a slightly bitter or metallic aftertaste. It is available as a tablet, powder, or liquid form and is widely used in food products including diet sodas, pharmaceuticals, and cosmetics. Saccharin has been approved for use in more than 100 countries world-wide. In high doses, saccharin has been found to be associated with an increase frequency of bladder cancer in a strain of male rats. However, a relationship between saccharin consumption and health risk in humans, at normal consumption amounts, has not been demonstrated and it is not considered to be a carcinogen [24].

### 2.2. Aspartame

 Aspartame was initially approved by the FDA in 1981 for limited use as a table top sweetener and for use in breakfast cereals, gelatins, and puddings. But in 1983, approval was extended to a larger group of food agents, including carbonated beverages. In 1996, the FDA approved aspartame as a general purpose sweetener for use in all foods and beverages. Aspartame is the most widely utilized non-cariogenic artificial sweetener and is 160–220 times more sweet than sucrose [25]. It is often the manufacturer's sweetener of choice in formulation of diet soft drinks, yogurt, puddings, gelatin, and snack foods. Prior to approval of aspartame by the FDA, a number of significant issues were raised by concerned individuals relative to aspartame's potential undesirable effects with long term consumption on growth, glucose homeostasis, neurotoxic effects in animals, behavioral reactions, seizure susceptibility and liver functions but these concerns have been largely addressed [26–31]. Aspartame is claimed to be safe for type 2 diabetics but should be avoided by people with phenylketonuria as they cannot metabolize phenylalanine, a component of aspartame. 

### 2.3. Acesulfame Potassium (Acesulfame K, Ace K)

 Acesulfame potassium, a calorie-free, non-cariogenic, nonnutritive artificial sweetener, was initially approved by the FDA in 1988 for use as a sweetener in dry food products. In 1994, yogurt, refrigerated desserts, syrups, and baked goods were added to the approved list, and in 2002 it was accepted as a general purpose sweetener. Acesulfame potassium is approximately 200 times sweeter than sucrose. More than thirty countries have approved the product to be used in foods, beverages, cosmetics, and pharmaceutical products. Like saccharin, acesulfame potassium, has a slightly bitter aftertaste and is often blended with other sweeteners to mask this property. Acesulfame potassium is stable at high cooking/baking temperatures, even under moderately acidic or basic conditions, which permits it to be used in baking or in products requiring a long shelf life. Although considered safe by the FDA for general consumption in food, there have been some concerns raised relative to dose-dependent cytogenetic toxicity [32, 33].

### 2.4. Sucralose

 Sucralose is a nonnutritive, noncaloric trichlorinated derivative of sucrose. It was first accepted by the FDA as an eating table-top sweetener in 1998 and followed by acceptance as a general purpose sweetener in 1999. It is 600 times sweeter than sucrose but is not metabolized by the body. Sucralose is considered safe for use by diabetics and has been shown not to be metabolized into acids by oral microbiota. It is heat stable during cooking and baking and is widely used in many food products such carbonated and noncarbonated beverages, as a tea and coffee sweetener, and in baked goods, chewing gum and frozen desserts. To date no health issues have been established concerning the general dietary use of sucralose [34, 35].

### 2.5. Neotame

 Neotame is a relatively recent approved noncaloric food product. It received FDA approval in 2002 for use as a general purpose sweetener in selected food products (except not in meat and poultry) and flavor enhancer. Neotame is an intense nonnutritive sweetener that is not fermentable by the oral microbiota and possesses a crisp, clean taste with no detectable aftertaste. It is reported to be greater than 7,000 times more potent than sucrose on a weight basis depending on the food product and how it is prepared [36, 37]. Neotame is a derivative of a dipeptide and has a similar chemical structure to aspartame. However, unlike aspartame, it is safe for consumption by people with phenylketonuria. It is also heat stable in baking applications and can be safely used by diabetics and pregnant women. Neotame is stable in carbonated soft drinks, powdered soft drinks, yellow cake, yogurt, and hot-packed still drinks [38]. 

## 3. FDA GRAS Approved Nonnutritive Sweeteners

**Figure 2:**
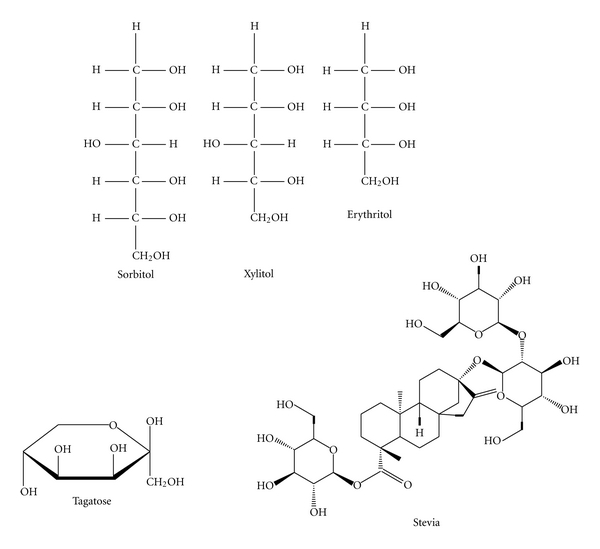
Chemical structure of FDA approved non-/low caloric sweeteners as GRAS (Generally Recognized as Safe).

 The FDA has approved as “Generally Recognized as Safe” (*GRAS*) several additional nonnutritive non-/low caloric calorie sweeteners, Figure 2.

### 3.1. Sorbitol

 Sorbitol is a 6-carbon sugar alcohol that occurs naturally in many fruits and berries. Although rather expensive to manufacturer, sorbitol is often used as a “bulk” sweetener in a variety of food substances such as chewing gum, chocolates, cakes and cookies, toothpaste, and mouthwash. On a weight basis, sorbitol is only half as sweet as sucrose. It is generally considered non-cariogenic, but sorbitol can be fermented slowly into acid by *S. mutans*. Research has shown sorbitol to possess mild cariogenic potential when used over an extended period of time by patients with reduced salivary gland function, and it normally supports the formation of dental plaque and the growth of mutans streptococci [39]. Specific remineralization-enhancing effect of sorbitol has not been shown [40]. It remains debatable among some authorities whether sorbitol should be consumed by diabetics. Sorbitol is not easily digested or absorbed from the gastrointestinal tract, and diarrhea is a potential side effect if ingested in large quantities [41].

### 3.2. Xylitol

 Xylitol, a five-carbon naturally occurring, nonfermentable, sugar alcohol, was first discovered in 1890 in birch and other hardwood tree chips, in wheat and oat straw in 1891, and later in various fruits and vegetables [42, 43]. It was approved by the FDA in 1986 for limited use. Xylitol is as sweet as sucrose and possesses a pleasant taste but is relatively expensive to manufacture. Although not as calorie heavy as sucrose, xylitol does possess a calorie burden when consumed and has some potential for increasing blood glucose. It is used primarily in mints, chewing gum, and toothpaste but is also available for table use. Studies have suggested that the regular use of xylitol containing chewing gum reduces the quantity of dental plaque, significantly reduced *S. mutans* levels, and increases saliva production [44, 45]. Reduction of caries incidence and remineralization of caries lesions have been reported in caries susceptible individuals when chewing gum containing xylitol was regularly used [46–50]. Xylitol has also been shown to inhibit cytokine expression by a lipopolysaccharide from one of the suspected periodontal pathogen bacteria, *Porphyromonas gingivalis *[51]. Thus, its regular use could possibly aid in preventing periodontal disease and gingival inflammation. Xylitol has been credited in lowering the risk of cariogenic bacteria transmission from mother to infant when compared to chlorhexadine and fluoride varnish treatments and reducing the incidence of ear infections among children at day-care centers [52–55]. However, excessive use of xylitol can aggravate symptoms of Crohn's disease and irritable bowel syndrome resulting in diarrhea [56, 57].

### 3.3. Erythritol

 Erythritol, a four-carbon sugar alcohol, has similar characteristics of sorbitol, mannitol, and xylitol. It is manufactured by a process that begins with fermenting glucose. But, it is only slightly more than half as sweet (70%) as sucrose and does not dissolve in water as well, but has significantly fewer calories by weight (0.2 calories per gram versus 4 calories per gram). Erythritol has been used in Japan since 1990 as a component of candies, soft drinks, chewing gum, jams, and yogurt. It was given *GRAS *recognition by the FDA in 1997. Erythritol is heat stable and can be used in baking and as a sweetener in low carbohydrate/calorie diets. It is almost completely absorbed by the small intestine (and excreted unchanged in the urine within 24 hours), has shown no toxic or carcinogenic effects, and is considered safe for consumption by diabetics. No long-term human caries trial on erythritol has been completed. However, the daily use of erythritol has been shown to reduce mutans streptococci levels in plaque and saliva [58]. Erythritol does not cause bloating, flatulence, or diarrhea at normal consumptions levels but may have a laxative effect in both children and adults if consumed in excess [59].

### 3.4. Tagatose

 Tagatose, a low-calorie natural sugar, has all the good qualities of erythritol plus it has about the same (92%) sweetness as sucrose, performs better in cooking, and has been shown to actually improve blood sugar control in diabetics. It has about 1/3 the number of calories as sucrose by weight. It was granted *GRAS* status in 2001 and is used in a variety of drugs, foods such as chocolates, chewing gum, cakes, ice cream and frosted cereals, beverages, and dietary supplements. Tagatose has been shown to have benefits treating noninsulin-dependent type 2 diabetes as it attenuates the rise of serum glucose after oral glucose intake [60]. No significant adverse health effects have been associated with the ingestion of this product when consumed in reasonable amounts. Excessive consumption can lead to mild intestinal discomfort, flatulence, and diarrhea [61].

### 3.5. Stevia

 Stevia, a heat stable sweetener with little or no aftertaste, is an extract from the herb *Stevia rebaudiana *Bertoni [62]. The extracted active ingredient is a white crystalline material. Its sweetness potency is many times greater (200–300) than sucrose. Stevia is calorie-free and non-cariogenic. The herb is native to Central and South America and has been used by the indigenous peoples of this area for centuries as a sweetener [63]. It has been used extensively in China, Brazil, and Japan, and to a lesser extent in Germany, Malaysia, and Israel, for many years as a sweetener in numerous food categories [64]. Originally banned by the FDA, the use of stevia was approved in 1995, as a *dietary supplement* but not as an *additive*. The argument to approve stevia as a *food additive* was heated, and it remained approved only as a *food supplement *for an extended period of time. However, in December 2008, the FDA responded favorably to GRAS status for the chemically refined derivative of stevia, the extract Rebaudioside A (Rebiana), to be used as a general purpose sweetener [65]. Rebiana is also available in combination with dextrose and as an extract from stevia leaves. Stevia has been shown to be safe for use by diabetics and has not been shown to be mutagenic [66, 67].

## 4. Discussion

 New nonnutritive sweeteners have been introduced into human diets over the past few decades. Oral health care professionals are often called upon to provide knowledgeable advice regarding the importance of diet and the role of sugars and nonnutritive sweeteners in caries formation and weight control. As such, they must be familiar with alternatives to sugar and the types of food products that are available with substitute non-/low caloric, non-cariogenic sweetening agents. An excellent literature review of the caries incidence and remineralization properties of the sugar alcohols (xylitol, erythritol, sorbitol) has been written by Mäkinen [68]. Although nonnutritive sweeteners do not generally promote dental caries, a program to prevent dental decay and promote oral health must also include good oral hygiene habits, regular dental professional care, and exposure to fluoride [69, 70]. Whether the use of nonnutritive sweeteners has a positive impact on weight loss by consumers remains controversial [71–75]. It has been postulated that nonnutritive sweeteners encourage sugar craving and dependency because of their sweet nature, and flavor preference occurs with repeated exposures to sweet tasting foods and beverages [76]. Several studies have shown an increase in BMI with consumption of nonnutritive sweeteners [77, 78]. But, others have found the evidence less compelling and more equivocal [79–81]. Whether nonnutritive sweetener use has a role in the current obesity and diabetes epidemic, whether beneficial, neutral, or not remains undetermined. In addition, consumption of two or more servings of nonnutritive sweetened sodas has been associated with a 2-fold increased odds for kidney function decline in women as measured by the eGFR (estimated Glomerular Filtration Rate) [82]. However, it is well established that a reduction of fermentable sugars and carbohydrates in the diet coupled with good oral hygiene practices will reduce the incidence of dental decay.

 While it is difficult to totally avoid sugar in the diet, as it is often added to processed food to enhance the taste, reducing the amount and frequency of dietary exposure to sugar is an important adjunct in preventing caries and reducing calorie intake although not without some potential health concerns as previously described [83–85]. However, nonnutritive sweeteners offer an attractive alternative to sugar in caries prevention and a possible adjunct in weight control when used appropriately and in concert with a balanced diet and exercise [86]. The identification of safe, palatable, heat stable, non-/low-caloric, nonnutritive/non-cariogenic sweetener substitutes for the more dental decay promoting and calorie heavy sugars such as sucrose, glucose, fructose, and maltose continues to be actively pursued.

 In addition to annually updating the health history, dental professionals should determine annually the BMI percentile of their patients and refer those on unhealthy trajectories to their physician or a dietitian for additional counseling [20]. It also behooves the dental professional to stay attuned to current information relative to alternative sweetener products that exist or are being developed and approved for dietary consumption by the FDA and be prepared to be a source of counseling for their patients and families as they relate to reducing the incidence of caries and possible overweight [87, 88].

## References

[1] Center for Disease Control and Prevention National Oral Heal Surveillance System. http://www.cdc.gov/nohss.

[2] Ogden CL, Yanovski SZ, Carroll MD, Flegal KM (2007). The epidemiology of obesity. *Gastroenterology*.

[3] Wang Y, Beydoun MA (2007). The obesity epidemic in the United States—gender, age, socioeconomic, racial/ethnic, and geographic characteristics: a systematic review and meta-regression analysis. *Epidemiologic Reviews*.

[4] Jensen ME (1999). Diet and dental caries. *Dental clinics of North America*.

[5] Gustafsson BE, Quensel CE, Lanke LS (1954). The Vipeholm dental caries study; the effect of different levels of carbohydrate intake on caries activity in 436 individuals observed for five years. *Acta odontologica Scandinavica*.

[6] Scheinin A, Mäkinen KK (1976). Turku sugar studies. An overview. *Acta Odontologica Scandinavica*.

[7] American Academy of Pediatrics Committee on Nutrition (2003). Policy statement on prevention of overweight and obesity. *Pediatrics*.

[8] Centers for Disease Control, Centers for Disease Control and Prevention Overweight and Obesity. Defining overweight and obesity. http://www.cdc.gov/obesity/defining.html.

[9] Johnson RK, Frary C (2001). Choose beverages and foods to moderate your intake of sugars: The 2000 Dietary Guidelines for Americans—what’s all the fuss about?. *Journal of Nutrition*.

[10] Kant AK (2000). Consumption of energy-dense, nutrient-poor foods by adult Americans: nutritional and health implications. The third National Health and Nutrition Examination Survey, 1988–1994. *American Journal of Clinical Nutrition*.

[11] National Academy of Sciences and Institute of Medicine (2005). *Dietary References Intakes for Energy, Carbohydrate, Fiber, Fat, Fatty Acids, Cholesterol, Protein and Amino Acids*.

[12] Tseng R, Vann WF, Perrin EM (2010). Addressing childhood overweight and obesity in the dental office: rationale and practical guidelines. *Pediatric Dentistry*.

[13] Harel-Raviv M, Laskaris M, Chu KS (1996). Dental caries and sugar consumption into the 21st century. *American Journal of Dentistry*.

[14] Rugg-Gunn AJ, Murray JJ (1989). Diet and dental caries. *The Prevention of Dental Diseases*.

[15] Scheinin A, Makinen KK (1975). Turku sugar studies I-XXI. *Acta Odontologica Scandinavica*.

[16] Koulourides T, Bodden R, Keller S (1976). Cariogenicity of nine sugars tested with an intraoral device in man. *Caries Research*.

[17] Kandelman D (1997). Sugar, alternative sweeteners and meal frequency in relation to caries prevention: new perspectives. *British Journal of Nutrition*.

[18] Sreebny LM (1982). Sugar and human dental caries. *World review of nutrition and dietetics*.

[19] Burt BA (1993). Relative consumption of sucrose and other sugars: has it been a factor in reduced caries experience?. *Caries Research*.

[20] Reilly JJ, Dorosty AR, Emmett PM (2000). Identification of the obese child: adequacy of the body mass index for clinical practice and epidemiology. *International Journal of Obesity*.

[21] (1998). Clinical guidelines on the identification, evaluation, and treatment of overweight and obesity in adults: The evidence report. National Institutes of Health. *Obesity Research*.

[22] Ogden CL, Kuczmarski RJ, Flegal KM (2002). Centers for Disease Control and Prevention 2000 growth charts for the United States: improvements to the 1977 National Center for Health Statistics version. *Pediatrics*.

[23] Braithwaite AS, Vann WF, Switzer BR, Boyd KL, Lee J (2008). Scientific article nutritional counseling practices: how do north carolina pediatric dentists weigh in?. *Pediatric Dentistry*.

[24] Weihrauch MR, Diehl V (2004). Artificial sweeteners—do they bear a carcinogenic risk?. *Annals of Oncology*.

[25] Kinghorn AD, Kaneda N, Baek NI, Kennelly EJ, Soejarto DD (1998). Noncariogenic intense natural sweeteners. *Medicinal Research Reviews*.

[26] Goerss AL, Wagner GC, Hill WL (2000). Acute effects of aspartame on aggression and neurochemistry of rats. *Life Sciences*.

[27] Wurtman RJ (1983). Neurochemical changes following high-dose aspartame with dietary carbohydrates. *New England Journal of Medicine*.

[28] Maher TJ, Wurtman RJ (1987). Possible neurologic effects of aspartame, a widely used food additive. *Environmental Health Perspectives*.

[29] Olney JW, Farber NB, Spitznagel E, Robins LN (1996). Increasing brain tumor rates: is there a link to aspartame?. *Journal of Neuropathology and Experimental Neurology*.

[30] National Cancer Institute Fact Sheet, Artificial Sweeteners and Cancer. http://www.cancer.gov/cancertopics/factsheet/Risk/artificial-sweeteners.

[31] Council on Scientific Affairs (1985). Aspartame: review of safety issues. *Journal of the American Medical Association*.

[32] Mukherjee A, Chakrabarti J (1997). In vivo cytogenetic studies on mice exposed to acesulfame-K—a non-nutritive sweetener. *Food and Chemical Toxicology*.

[33] Kroger M, Meister K, Kava R (2006). Low-calorie sweeteners and other sugar substitutes: a review of the safety issues. *Comprehensive Reviews in Food Science and Food Safety*.

[34] (1998). *Federal Register*.

[35] Grice HC, Goldsmith LA (2000). Sucralose—an overview of the toxicity data. *Food and Chemical Toxicology*.

[36] Walters DE, Prakash I, Desai N (2000). Active conformations of neotame and other high-potency sweeteners. *Journal of Medicinal Chemistry*.

[37] Prakash I, Bishay IE, Desai N, Eric Walters D (2001). Modifying the temporal profile of the high-potency sweetener neotame. *Journal of Agricultural and Food Chemistry*.

[38] Witt J (1999). Discovery and development of neotame. *World Review of Nutrition and Dietetics*.

[39] Wennerholm K, Arends J, Birkhed D, Ruben J, Emilson CG, Dijkman AG (1994). Effect of xylitol and sorbitol in chewing-gums on mutans streptococci, plaque pH and mineral loss of enamel. *Caries research*.

[40] Creanor SL, Strang R, Gilmour WH (1992). The effect of chewing gum use on in situ enamel lesion remineralization. *Journal of Dental Research*.

[41] Hyams JS (1983). Sorbitol intolerance: an unappreciated cause of functional gastrointestinal complaints. *Gastroenterology*.

[42] Bertrand MG (1891). Rechercheszur quelques derives du xylose. *Bulletin de la Société Chimique de France*.

[43] Fischer E, Stahel R (1891). Zur kenntnis der xylose. *Berichte der Deutschen Chemischen Gesellschaft*.

[44] Hildebrandt GH, Sparks BS (2000). Maintaining mutans streptococci suppression: with xylitol chewing gum. *Journal of the American Dental Association*.

[45] Isokangas P, Tenovuo J, Söderling E, Männistö H, Mäkinen KK (1991). Dental caries and mutans streptococci in the proximal areas of molars affected by the habitual use of xylitol chewing gum. *Caries Research*.

[46] Isokangas P, Alanen P, Tiekso J, Makinen KK (1988). Xylitol chewing gum in caries prevention: a field study in children. *The Journal of the American Dental Association*.

[47] Mäkinen KK, Bennett CA, Hujoel PP (1995). Xylitol chewing gums and caries rates: a 40-month cohort study. *Journal of Dental Research*.

[48] Autio JT (2002). Effect of xylitol chewing gum on salivary streptococcus mutans in preschool children. *Journal of Dentistry for Children*.

[49] Hayes C (2001). The effect of non-cariogenic sweeteners on the prevention of dental caries: a review of the evidence. *Journal of dental education*.

[50] Mäkinen KK, Mäkinen PL, Pape HR (1996). Conclusion and review of the ’Michigan Xylitol Programme’ (1986–1995) for the prevention of dental caries. *International Dental Journal*.

[51] Han SJ, Jeong SY, Nam YJ, Yang KH, Lim HS, Chung J (2005). Xylitol inhibits inflammatory cytokine expression induced by lipopolysaccharide from *Porphyromonas gingivalis*. *Clinical and Diagnostic Laboratory Immunology*.

[52] Mäkinen KK (2000). The rocky road of xylitol to its clinical application. *Journal of Dental Research*.

[53] Mäkinen KK, Isotupa KP, Kivilompolo T, Mäkinen PL, Toivanen J, Söderling E (2001). Comparison of erythritol and xylitol saliva stimulants in the control of dental plaque and mutans streptococci. *Caries Research*.

[54] Uhari M, Kontiokari T, Koskela M, Niemelä M (1996). Xylitol chewing gum in prevention of acute otitis media: double blind randomised trial. *British Medical Journal*.

[55] Söderling E, Isokangas P, Pienihäkkinen K, Tenovuo J, Alanen P (2001). Influence of maternal xylitol consumption on mother-child transmission of mutans streptococci: 6-year follow-up. *Caries Research*.

[56] Newberne PM, Conner MW, Estes P (1988). The influence of food additives and related materials on lower bowel structure and function. *Toxicologic Pathology*.

[57] Ludwig B, Schindler E, Bohl J (1984). Reno-cerebral oxalosis induced by xylitol. *Neuroradiology*.

[58] Mäkinen KK, Saag M, Isotupa KP (2005). Similarity of the effects of erythritol and xylitol on some risk factors of dental caries. *Caries Research*.

[59] eHOW What is Erythritol Sweetener?. http://www.ehow.com/facts_5028505_erythritol-sweetener.html.

[60] Donner TW, Wilber JF, Ostrowski D (1999). D-tagatose, a novel hexose: acute effects on carbohydrate tolerance in subjects with and without type 2 diabetes. *Diabetes, Obesity and Metabolism*.

[61] Levin GV (2002). Tagatose, the new GRAS sweetener and health product. *Journal of Medicinal Food*.

[62] Cardello HMAB, Da Silva MAPA, Damasio MH (1999). Measurement of the relative sweetness of stevia extract, aspartame and cyclamate/saccharin blend as compared to sucrose at different concentrations. *Plant Foods for Human Nutrition*.

[63] Lewis WH (1982). Early uses of stevia rebaudiana (Asteraceae) leaves as a sweetener in Paraguay. *Economic Botany*.

[64] Nabors LO, Gelardi RC (1991). *Alternative Sweeteners*.

[65] http://www.accessdata.fda.gov/scripts/fcn/fcnDetailNavigation.cfm?rpt=grasListing&id=252.

[66] Chan P, Tomlinson B, Chen YJ, Liu JC, Hsieh MH, Cheng JT (2000). A double-blind placebo-controlled study of the effectiveness and tolerability of oral stevioside in human hypertension. *British Journal of Clinical Pharmacology*.

[67] Matsui M, Matsui K, Kawasaki Y (1996). Evaluation of the genotoxicity of stevioside and steviol using six in vitro and one in vivo mutagenicity assays. *Mutagenesis*.

[68] Mäkinen KK (2010). Sugar alcohols, caries incidence, and remineralization of caries lesions: a literature review. *International Journal of Dentistry*.

[69] Touger-Decker R, van Loveren C (2003). Sugars and dental caries. *The American Journal of Clinical Nutrition*.

[70] Navia JM (1994). Carbohydrates and dental health. *American Journal of Clinical Nutrition*.

[71] Mattes RD, Popkin BM (2009). Nonnutritive sweetener consumption in humans: effects on appetite and food intake and their putative mechanisms. *American Journal of Clinical Nutrition*.

[72] Brown RJ, De Banate MA, Rother KI (2010). Artificial sweeteners: a systematic review of metabolic effects in youth. *International Journal of Pediatric Obesity*.

[73] Yang Q (2010). Gain weight by "going diet?" Artificial sweeteners and the neurobiology of sugar cravings: neuroscience 2010. *Yale Journal of Biology and Medicine*.

[74] Vermunt SHF, Pasman WJ, Schaafsma G, Kardinaal AFM (2003). Effects of sugar intake on body weight: a review. *Obesity Reviews*.

[75] Drewnowski A (1995). Intense sweeteners and the control of appetite. *Nutrition Reviews*.

[76] Liem DG, De Graaf C (2004). Sweet and sour preferences in young children and adults: role of repeated exposure. *Physiology and Behavior*.

[77] Blum JW, Jacobsen DJ, Donnelly JE (2005). Beverage consumption patterns in elementary school aged children across a two-year period. *Journal of the American College of Nutrition*.

[78] Berkey CS, Rockett HRH, Field AE, Gillman MW, Colditz GA (2004). Sugar-added beverages and adolescent weight change. *Obesity Research*.

[79] Williams CL, Strobino BA, Brotanek J (2007). Weight control among obese adolescents: a pilot study. *International Journal of Food Sciences and Nutrition*.

[80] Striegel-Moore RH, Thompson D, Affenito SG (2006). Correlates of beverage intake in adolescent girls: The National Heart, Lung, and Blood Institute Growth and Health Study. *Journal of Pediatrics*.

[81] Benton D (2005). Can artificial sweeteners help control body weight and prevent obesity?. *Nutrition Research Reviews*.

[82] Lin J, Curhan GC (2011). Associations of sugar and artificially sweetened soda with albuminuria and kidney function decline in women. *Clinical Journal of the American Society of Nephrology*.

[83] Moynihan PJ (1998). Update on the nomenclature of carbohydrates and their dental effects. *Journal of Dentistry*.

[84] Raben A, Vasilaras TH, Christina Møller A, Astrup A (2002). Sucrose compared with artificial sweeteners: different effects on ad libitum food intake and body weight after 10 wk of supplementation in overweight subjects. *American Journal of Clinical Nutrition*.

[85] Blackburn GL, Kanders BS, Lavin PT, Keller SD, Whatley J (1997). The effect of aspartame as part of a multidisciplinary weight-control program on short- and long-term control of body weight. *American Journal of Clinical Nutrition*.

[86] Rethman MP, Beitrán-Aguilar ED, Billings RJ (2011). Nonfluoride caries-preventive agents: executive summary of evidence-based clinical recommendations. *Journal of the American Dental Association*.

[87] Roberts MW, Wright JT (2010). Sweetness without sugar. *Dimensions of Dental Hygiene*.

[88] Vann WF, Bouwens TJ, Braithwaite AS, Lee JY (2005). The childhood obesity epidemic a role for pediatric dentists?. *Pediatric Dentistry*.

